# Engineering Botulinum Neurotoxin C1 as a Molecular Vehicle for Intra-Neuronal Drug Delivery

**DOI:** 10.1038/srep42923

**Published:** 2017-02-21

**Authors:** Edwin J. Vazquez-Cintron, Phillip H. Beske, Luis Tenezaca, Bao Q. Tran, Jonathan M. Oyler, Elliot J. Glotfelty, Christopher A. Angeles, Aurelia Syngkon, Jean Mukherjee, Suzanne R. Kalb, Philip A. Band, Patrick M. McNutt, Charles B. Shoemaker, Konstantin Ichtchenko

**Affiliations:** 1 Department of Biochemistry and Molecular Pharmacology, New York University School of Medicine, New York, NY, 10016, USA; 2 CytoDel LLC, New York, NY, 10027, USA; 3 The United States Army Medical Research Institute of Chemical Defense, Aberdeen Proving Ground, MD 21010, USA; 4 Excet, Inc., 6225 Brandon Ave., Suite 360, Springfield, VA, 22150, USA; 5 Department of Infectious Diseases and Global Health, Tufts University Clinical and Translational Science Institute, North Grafton, MA, 01536, USA; 6 Centers for Disease Control and Prevention, National Center for Environmental Health/Agency for Toxic Substances and Disease Registry, Atlanta, GA 30341, USA; 7 Department of Orthopaedic Surgery, New York University Hospital for Joint Diseases, New York, NY, 10016, USA

## Abstract

Botulinum neurotoxin (BoNT) binds to and internalizes its light chain into presynaptic compartments with exquisite specificity. While the native toxin is extremely lethal, bioengineering of BoNT has the potential to eliminate toxicity without disrupting neuron-specific targeting, thereby creating a molecular vehicle capable of delivering therapeutic cargo into the neuronal cytosol. Building upon previous work, we have developed an ***a***toxic ***d***erivative (*ad*) of BoNT/C1 through rationally designed amino acid substitutions in the metalloprotease domain of wild type (*wt*) BoNT/C1. To test if BoNT/C1 *ad* retains neuron-specific targeting without concomitant toxic host responses, we evaluated the localization, activity, and toxicity of BoNT/C1 *ad in vitro* and *in vivo*. In neuronal cultures, BoNT/C1 *ad* light chain is rapidly internalized into presynaptic compartments, but does not cleave SNARE proteins nor impair spontaneous neurotransmitter release. In mice, systemic administration resulted in the specific co-localization of BoNT/C1 *ad* with diaphragmatic motor nerve terminals. The mouse LD_50_ of BoNT/C1 *ad* is 5 mg/kg, with transient neurological symptoms emerging at sub-lethal doses. Given the low toxicity and highly specific neuron-targeting properties of BoNT/C1 *ad*, these data suggest that BoNT/C1 *ad* can be useful as a molecular vehicle for drug delivery to the neuronal cytoplasm.

Delivery of biological moieties to the neuronal cytosol provides a means of treating neurological conditions that have been difficult to address by conventional therapies. However, the development of non-viral molecular vehicles that are capable of intracellular delivery to central and peripheral neurons remains a critical limitation. Botulinum BoNT has unique potential as a novel therapeutic vehicle for several reasons; the toxin recognizes the presynaptic membrane of neurons with exquisite specificity, its light chain is not sequestered in endosomes, and it has the ability to translocate from the lumen of synaptic endosomes to the presynaptic cytosol^1^. However, to exploit the potential of BoNT as a neuronal delivery vehicle, it must be bioengineered to eliminate its toxic properties without disrupting the ability to reach the presynaptic cytosol of neurons.

BoNT, the causative agent of botulism in humans, is produced by the anaerobic spore-forming Gram-positive bacterium *Clostridium botulinum* and related species. Using serological methods, seven different BoNT serotypes (labeled A-G) have been identified. The mature processed form of BoNT exists as a ~150 kDa disulfide-bonded heterodimer consisting of a light chain (LC, ~50 kDa) and heavy chain (HC, ~100 kDa). The heterodimer comprises three major functional domains: 1) the LC Zn^2+^-metalloprotease domain, which mediates toxicity through a series of precisely orchestrated events that occur following delivery to neuronal cytosol; 2) the HC C-terminal domain (H_C_, ~50 kDa), responsible for neuron-specific receptor binding at the presynaptic membrane; and 3) the HC N-terminal domain (H_N_, ~50 kDa), responsible for translocating the LC through the endosomal membrane and into the neuronal cytosol^2^,^3^,^4^. Intoxication with BoNT results in inhibition of pre-synaptic neurotransmitter release at the neuromuscular junction (NMJ). The toxic effect of BoNT is achieved through persistent cleavage of components of the **S**oluble **N**SF **A**ttachment Protein **RE**ceptor (SNARE) complex required for exocytosis of neurotransmitters^3^. Although there are differences among the serotypes in host range, receptor binding and the precise proteolytic target, productive intoxication by any serotype results in neuromuscular paralysis due to exocytotic blockade.

We have previously described genetic constructs and expression systems that enable facile design and production of atoxic recombinant derivatives of BoNT serotype A1 (BoNT/A1 *ad*) that retain the structural and trafficking properties of *wt* BoNT/A1^5^,^6^. One such derivative, BoNT/A1 *ad* was developed and described as a “Trojan horse” prototype molecular vehicle for delivering drugs to the neuronal cytoplasm. BoNT/A1 *ad* was rendered atoxic by introducing two amino acid substitutions in the active site of *wt* BoNT/A1 LC^5^,^6^,^7^. While BoNT/A1 *ad* was capable of delivering its LC to the pre-synaptic compartment of neurons at concentration of up to 1 nM, BoNT/A1 *ad* was only about 100,000 times less toxic than *wt* BoNT/A1, and therefore, BoNT/A1 ad suffered from a narrow therapeutic dosage window^7^.

Here, we describe the design, expression, purification, and functional evaluation of a second-generation neuron-specific delivery vehicle composed of an atoxic BoNT derivative with reduced toxicity that circumvents the limitations of the BoNT/A1 *ad*. This new delivery vehicle is a derivative of BoNT serotype C1 (BoNT/C1 *ad*) that was rendered atoxic by three substitutions in the LC. The toxicity and neuron-targeting properties of BoNT/C1 *ad* were evaluated *in vitro* and *in vivo*. BoNT/C1 *ad* LC was internalized into the neuronal cytosol, where it stably persisted for at least 8 days. BoNT/C1 *ad* trafficked to the presynaptic compartment where it co-localized with pre-synaptic proteins, with minor co-localization with endosomal and lysosomal markers. Treatment of neuronal cultures with BoNT/C1 *ad* did not result in detectable cleavage of SNARE proteins or cytotoxicity, even at concentrations that showed toxicity symptoms *in vivo*. In an *in vivo* mouse model, BoNT/C1 *ad* had significantly lower toxicity than BoNT/A1 *ad*, and rapidly accumulated at the NMJ in the diaphragm. The extremely low *in vivo* toxicity of BoNT/C1 *ad* and its neuron-targeting properties suggest that it will be useful as a molecular vehicle for drug delivery to the neuronal cytoplasm.

## Results

### Potency of wt BoNT/C1 batches used in this study

We found differences in potencies as high as 31-fold among the *wt* BoNT/C1 preparations used in our study. Therefore, in presenting our data, we elected to provide values for *wt* BoNT/C1 as both molar concentration (when we needed to accent the difference in concentrations between *wt* BoNT/C1 and BoNT/C1 *ad*) and LD_50_ (expressed as mouse LD_50_ units or mouse LD_50_ units/mL). We also provide both values for BoNT/C1 *ad*.

### BoNT/C1 ad design, expression, and purification

**Figure 1:**
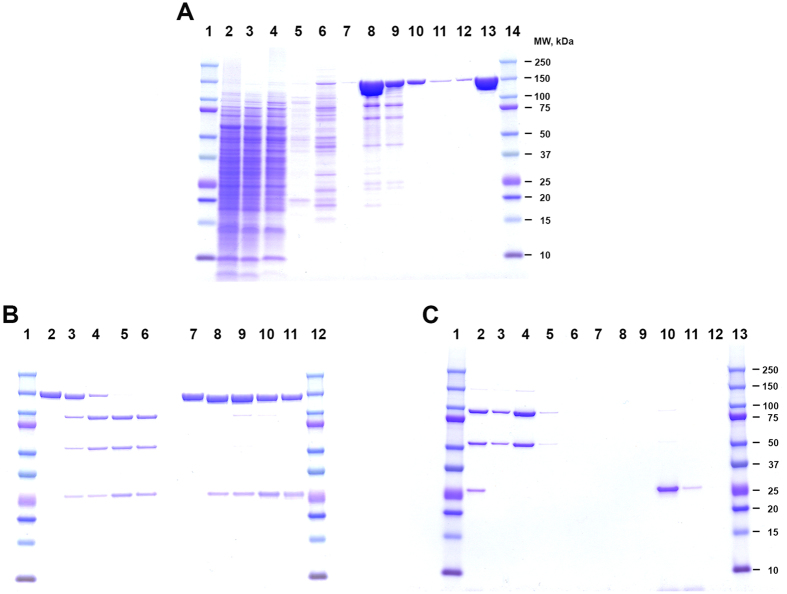
BoNT/C1 *ad* expression, purification, and processing. (**A**) Coomassie blue BB R250 stained 4–12% SDS PAGE analysis of each step in BoNT/C1 *ad* pro-peptide purification by tandem affinity chromatography: lanes 1 and 14 – protein MW ladder; lanes 2–8 – products of steps of Ni^2+^-NTA affinity chromatography: lane 2 – loading material: concentrated and dialyzed culture supernatant containing BoNT/C1 *ad* pro-peptide; lane 3 – flow through; lane 4 – first wash with 15 mM imidazole buffer; lane 5 – second wash with 15 mM imidazole buffer; lane 6 – first wash with 45 mM imidazole buffer; lane 7 – second wash with 45 mM imidazole buffer; lane 8 – eluate obtained with 250 mM imidazole buffer; lanes 9–12 – steps of StrepTactin affinity chromatography of eluate from lane 8; lane 9 – flow through; lanes 10 and 11 – sequential washes with high (1 M NaCl) salt buffer; lane 12 – wash with low salt buffer; lane 13 – eluate obtained with 5 mM *D-*desthiobiotin. (**B**) Processing of BoNT/C1 *ad* pro-peptide to heterodimer by proteolytic cleavage with TEV protease: lanes 1 and 12 – protein MW ladder; lanes 2–6 – samples reduced by incubation with β-mercaptoethanol; lanes 7–11 – non-reduced samples; lanes 2 and 7 – no TEV protease added; lanes 3 and 8 – BoNT/C1 *ad* pro-peptide incubated with TEV protease for 1 hour at 25 °C; lanes 4 and 9 – incubated with TEV protease for six hours; lanes 5 and 10 – incubated with TEV protease for 24 hours; lanes 6 and 11 – incubated with TEV protease for 48 hours. (**C**) Removal of TEV protease from the reaction mixture by Ni^2+^-NTA affinity chromatography: lanes 1 and 13 – protein MW ladder; lane 2 – loading material; lanes 3–5 – sequential washes with 15 mM imidazole buffer; lanes 6–9 – sequential washes with 45 mM imidazole buffer; lanes 10–12 – sequential elution with 250 mM imidazole buffer.

The LC of BoNT/C1 *ad* differs from *wt* BoNT/C1 as a result of three amino acid substitutions (E_238_ > A; H_241_ > G; Y_383_ > A). These substitutions were designed to inactivate the light chain metalloprotease with minimal disruption to light chain/heavy chain interactions within the protein heterodimer. The three amino acid residues selected for mutation in BoNT/C1 *ad* are 100% conserved among seven different BoNT LC serotypes; these amino acids were selected based on similar mutations described in our previous work with *wt* BoNT/A1^5^,^7^,^8^. To increase the yield of expressed protein, the DNA sequence encoding the full-length BoNT/C1 *ad* was synthesized *de novo* and optimized for expression in *Sf9* cells^5^. Major steps of protein expression and processing are shown in Fig. 1. BoNT/C1 *ad* was engineered with three peptide tags to facilitate purification and detection. The pro-peptide has a polyhistidine tag at the N-terminus, followed by a Tobacco Etch Virus (TEV) recognition sequence. A second TEV recognition sequence was incorporated between the C-terminus of the LC and the N-terminus of the HC. Finally, a hemagglutinin (HA) tag was placed at the C-terminus of the HC, followed by a third TEV recognition site and three *Strep* tag II repeats. The polyhistidine and *Strep* tag II repeats allow for two-step tandem affinity chromatography purification, and the HA tag enables specific immunodetection of the recombinant BoNT/C1 *ad* but not *wt* BoNT/C1. Following baculovirus-mediated expression in *Sf9* cells, the BoNT/C1 *ad* pro-peptide was sequentially purified by tandem affinity chromatography on Ni^2+^-NTA and StrepTactin Sepharose fast flow resins. For generation of the BoNT/C1 *ad* heterodimer and removal of purification tags, the purified pro-peptide was cleaved by treatment with TEV protease. Pilot-scale production of BoNT/C1 *ad* yielded approximately 50 mg of sterile pyrogen-free heterodimer per L of culture.

### Proteomic characterization of BoNT/C1 *ad* heterodimer

**Figure 2:**
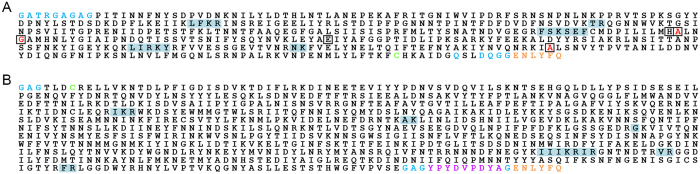
Proteomic characterization of BoNT/C1 *ad* heterodimer. (**A**) light chain sequence; (**B**) heavy chain sequence. Letters on white background represent confirmed sequence identified among one or more corresponding peptides from enzymatic digests of the recombinant protein. Letters on light blue background represent sequences not recovered from any of the digests. Blue letters represent exogenous linkers/spacers introduced into the recombinant protein. Boxed letters in Panel **A** mark positions of five non-adjacent amino acids – *H, E, H, E, Y* – that form the active site of Zn^2+^-metalloprotease; red letters among this group represent mutations E_238_ > A; H_241_ > G; Y_383_ > A introduced into the metalloprotease active site rendering it inactive in this atoxic derivative. Orange letters represent the TEV protease recognition sequence. Green letters represent the two cysteine residues that form the disulfide bridge between light and heavy chains of the heterodimer. Magenta letters (Panel **B**) represent the HA tag introduced at the C-terminus of the heavy chain for detection purposes.

Sequence fidelity of purified BoNT/C1 *ad* was confirmed by two methods: 1) Mass-spectrometry analysis (Fig. 2); and 2) Western blot using monoclonal antibodies against the HC, LC, and HA tag (Supplemental Figure S1). Mass spectrometry of the heterodimer by liquid chromatography and high-resolution tandem mass-spectrometry resulted in 96% and 98% sequence coverage for the light and heavy chains, respectively. Importantly, light chain peptides containing the mutation sites were identified, and point mutations were confirmed at E_238_ > A, H_241_ > G, and Y_383_ > A (Fig. 2A). The recombinant linkers and spacers, the TEV protease recognition sequence, and the C-terminal HA tag were also identified. The missing sequence regions were less than 5 residues in length and were likely excluded from MS data acquisition as well as database searching because of their relative lack of specificity.

### BoNT/C1 *ad* murine median lethal dose

The standard method of defining the potency or toxicity of BoNTs is by determination of the median lethal dose (LD_50_) following intraperitoneal (*ip*) injection^9^. To determine the LD_50_ of BoNT/C1 *ad* in mice, animals were administered doses of BoNT/C1 *ad* from 0.04 to 6.00 mg/kg (Table 1). The LD_50_ of BoNT/C1 *ad* was determined to be 5 mg/kg by two independent laboratories, while mice treated with 2 mg/kg or greater showed clinical symptoms consistent with neuromuscular impairment, including wasp-like waist, generalized body weakness, and altered respiratory pattern (Table 1). Mice that developed adverse effects and survived were clinically asymptomatic by 120 hours after injection. In comparison, the intraperitoneal LD_50_ of a “standard” potency batch of *wt* BoNT/C1 is reported to be approximately 1 ng/kg^10^. These data suggest that the recombinant atoxic derivative is approximately 5 × 10^6^-fold less toxic than its wild type precursor.

### BoNT/C1 *ad* is catalytically inactive

**Figure 3:**
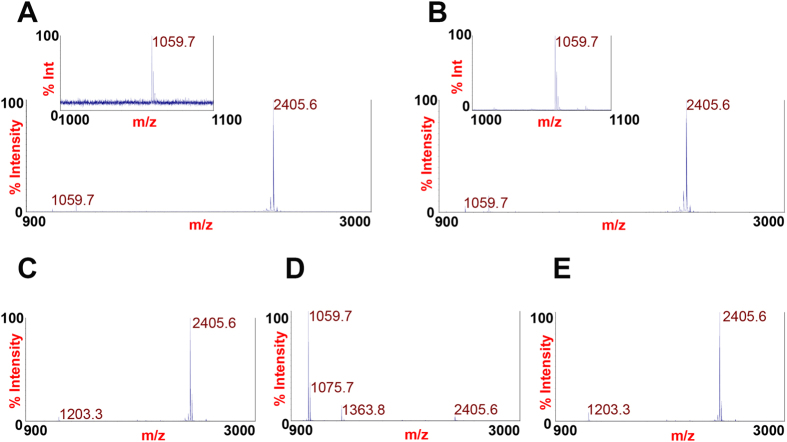
BoNT/C1 *ad* is catalytically inactive. MS analysis of cleavage products. Substrate for *wt* BoNT/C1 (SubC) was incubated with 18.4 pg *wt* BoNT/C1, corresponding to 1 mouse LD_50_ unit (Panel **A**); 184 pg of *wt* BoNT/C1, corresponding to 10 mouse LD_50_ units (Panel **B**); vehicle (Panel **C**); 100 ng of *wt* BoNT/C1, corresponding to 5,435 mouse LD_50_ units (Panel **D**); or 10,000 ng of BoNT/C1 *ad*, corresponding to 6.7 mouse LD_50_ units (Panel **E**). Insets show m/z 1059.7. A peak representing un-cleaved substrate can be seen on all panels at m/z 2405.6. Peaks at m/z 1203.3 seen on Panels **A**, **B**, **C**, and **E** represent a doubly-charged form of the intact substrate (m/z 2405.6).

The cellular target of *wt* BoNT/C1 is the presynaptic compartment, where it associates with neuronal SNARE proteins and cleaves **S**y**n**aptosomal-**A**ssociated **P**rotein 25 (SNAP-25) and syntaxin-1 (Syx1). To confirm that the active site amino acid substitutions eliminated light chain enzymatic activity, mass-spectrometry studies were performed using a metalloprotease activity-based assay developed at the CDC^11^. This assay tests for the cleavage of a short *wt* BoNT/C1 substrate (SubC) using a highly sensitive mass spectrometry platform that can detect cleavage of substrate containing as little as 1 mouse LD_50_ of the toxin. Incubation of SubC with *wt* BoNT/C1 (18.4 pg, equivalent to 1 mouse LD_50_; 184 pg, equivalent to 10 mouse LD_50_, and 100 ng, equivalent to 5,435 mouse LD_50_) resulted in the proteolytic conversion of intact SubC (m/z = 2405.6) to peaks at m/z 1059.7 and 1363.8 (Fig. 3A, B and D). In contrast, incubation of SubC with 10,000 ng of BoNT/C1 *ad* (equivalent to 6.7 mouse LD_50_) did not result in cleavage, indicating that BoNT/C1 *ad* is catalytically inactive (Fig. 3E). These data indicate that the residual catalytic activity of BoNT/C1 *ad* is reduced by at least 543,478-fold in comparison to *wt* BoNT/C1. Notably, no evidence of SubC cleavage was detected when BoNT/C1 *ad* was used at concentrations corresponding to 6.7 mouse LD_50_ doses, suggesting that *in vivo* symptoms are not attributable to proteolytic activity.

**Figure 4:**
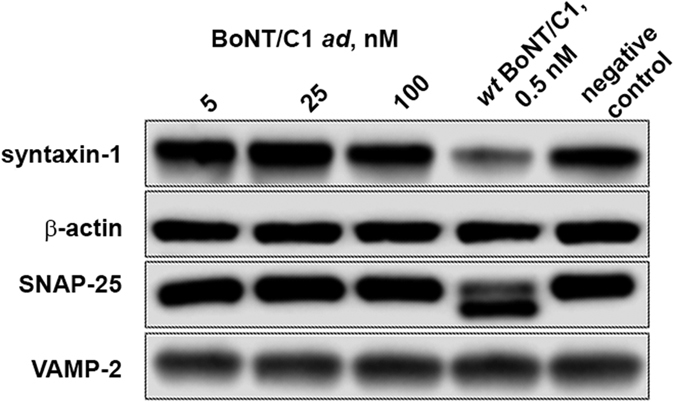
Cell-based assay of BoNT/C1 *ad* cleavage of SNAP-25 or Syx1. Primary cortical neurons maintained for 14 days after plating were exposed to BoNT/C1 *ad, wt* BoNT/C1, or medium alone for 96 hours, and analyzed by Western blotting. lane 1 – neurons were treated with BoNT/C1 *ad* (5 nM, equivalent to 0.01 mouse LD_50_ units/mL); lane 2 – neurons were treated with BoNT/C1 *ad* (25 nM, equivalent to 0.05 mouse LD_50_ units/mL), lane 3 – neurons were treated with BoNT/C1 *ad* (100 nM, equivalent to 0.2 mouse LD_50_ units/mL), lane 4 – neurons were treated with *wt* BoNT/C1 (0.5 nM, equivalent to 405 mouse LD_50_ units/mL) (positive control), lane 5 – neurons were treated with medium alone (negative control). Immunoblots were probed with mAbs against SNAP-25 and Syx1. Immunostaining with mAbs against VAMP-2 and β-actin were used as loading controls. Western blots have been cropped to only show bands visualized in each independent experiment.

To confirm that BoNT/C1 *ad* was catalytically inactive in a cellular environment, we treated 14-day E19 rat cortical neuron cultures with 5, 25, and 100 nM BoNT/C1 *ad* (equivalent to 0.01, 0.05, and 0.2 mouse LD_50_ units/mL of culture medium, respectively) or with an amount of *wt* BoNT/C1 equivalent to 405 mouse LD_50_ units per mL for 96 h, and assessed integrity of SNAP-25 and Syx1 by immunoblot analysis. Cultures treated with *wt* BoNT/C1 exhibited a loss of Syx1 immunoreactivity, and displayed a lower molecular weight band indicative of cleaved SNAP-25 (Fig. 4). In contrast, cultures treated with up to 100 nM BoNT/C1 *ad* exhibited no detectable cleavage of SNAP-25 or Syx1.

### BoNT/C1 *ad* light chain persistence in primary neurons *in vitro*

**Figure 5:**
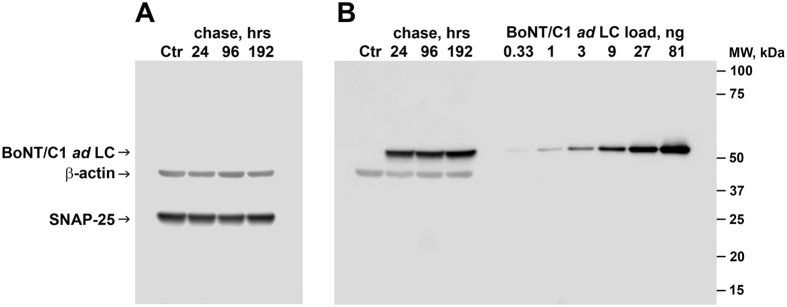
Stability of BoNT/C1 *ad* LC internalized by cortical neurons: Rat fetal E19 neurons were isolated, plated, treated with BoNT/C1 *ad*, harvested, and lysed. Total extracted protein was separated on reduced SDS PAGE, and analyzed by Western blot as described in Methods. The four lanes of Panel **A** and the first four lanes of Panel **B** represent identical samples in identical order. Panel **A** was probed with anti-β-actin and anti-SNAP-25 Mabs. Panel **B** was probed with anti- BoNT/C1 *ad* LC (4C.10.2) and anti-β-actin Mabs. Reduced BoNT/C1 samples corresponding to the amounts of BoNT/C1 *ad* LC shown on the top right of Panel **B** were used for quantification purposes. Molecular weight markers are shown on the right.

Experiments were performed to determine the persistence of the BoNT/C1 *ad* light chain in the cytoplasmic compartment of cortical neuronal cultures. Primary cortical neurons (14 day after plating) were treated with 25 nM BoNT/C1 *ad* for 48 h, and SNAP-25 integrity was evaluated by immunoblot 1, 4, or 8 days later. No cleavage of SNAP-25 was observed at any time point (Fig. 5A). BoNT/C1 *ad* LC was detected throughout the 8 days of chase (Fig. 5B). No evidence of light chain degradation was observed in any of the samples. The amount of BoNT/C1 *ad* LC recovered from the cells remained constant throughout the study. Based on densitometry using a standard curve of purified BoNT/C1 *ad*, we recovered 15.29 ± 6.49 ng (range: 8.8–21.78 ng) BoNT/C1 *ad* LC per 55 μg of total protein (Fig. 5B). Based on the number of viable cells per culture and estimates of cellular volume, the intracellular BoNT/C1 *ad* LC concentration was calculated to be 705 ± 95 nM or 1.41 ± 0.19 mouse LD_50_ units of BoNT/C1 *ad* per mL of neuronal cytoplasm (Table 2). Notably, no cleavage of the *wt* BoNT/C1 substrate, SNAP-25, was detected, even with a chase time up to 8 days in culture (Fig. 5A). This intracellular BoNT/C1 *ad* LC concentration exceeds the concentration of BoNT/C1 *ad* added to the medium (25 nM) by 28.2 ± 3.8 fold (range 32–24.4 fold), which suggests that the toxin was enriched within cells through a specific mechanism of internalization. In our hands, and consistent with other reports^12^, primary hippocampal neurons maintained in Neurobasal medium with B27 serum-free supplement started to exhibit a decline in neuronal survival after about 25 days in culture, and therefore, the total duration of these experiments was not extended beyond 24 days. These data indicate that BoNT/C1 *ad* LC accumulates within neurons and persists for at least 8 days with no detectable catalytic activity.

### Calculations of EC_50_ for the *wt* BoNT/C1 expressed as mouse LD_50_/mL

The EC_50_ (the concentration of toxin sufficient to cleave 50% of Syx1 and SNAP-25 in neurons treated for 24 hours) for the *wt* BoNT/C1 was previously determined to be 14.24 pM^13^. Since different batches of toxin have variable potencies, toxin is described in terms of mouse LD_50_ units/mL. One mouse LD_50_ unit for this batch of toxin was equal to 31.25 pg. The blood volume of an average 25 g mouse is equal to ~1,675 μL^14^; thus, the concentration of the protein after injection *in vivo* is equal to 31.25 pg/1,675 μL, or 18.7 fg /μL; whereas, 150 fg/μL represents a 1 pM toxin solution. Thus, 18.7 fg/μL represents a 125 fM solution. Therefore, a 14.24 pM solution is equal to 114 mouse LD_50_ units (14,240/125) per mL. We can not calculate an EC_50_ ratio for the BoNT/C1 *ad* because at all concentrations of BoNT/C1 *ad* used *in vitro* in this study, we could not detect either syntaxin-1 or SNAP-25 cleavage. However, the EC_50_ data suggested that the assay utilizing cleavage of cellular substrates, followed by Western blot detection was not very sensitive. In contrast to EC_50_ measurements, IC_50_ measurements (50% inhibition of neurotransmitter release) are 10–100 orders of magnitude more sensitive^15^,^16^. We used a similar approach (IC_80_) in the current work (next paragraph).

### Effect of BoNT/C1 *ad* on synaptic transmission in mESNs

**Figure 6:**
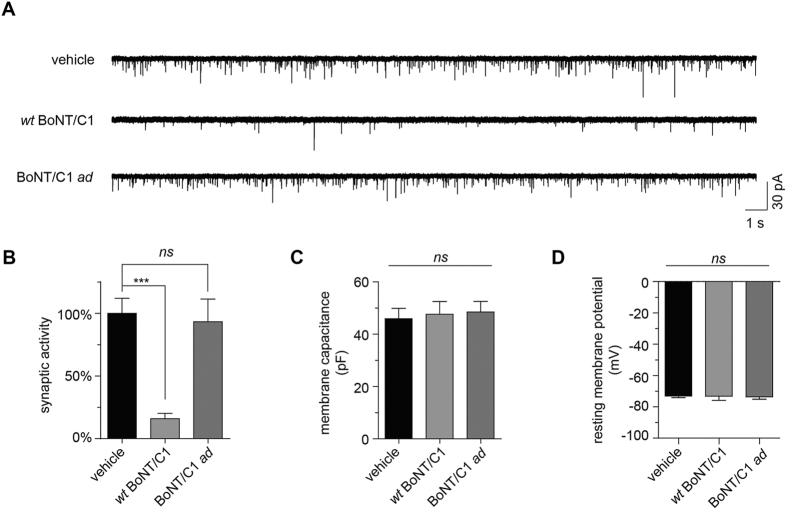
BoNT/C1 *ad* does not impair spontaneous pre-synaptic neurotransmitter release. (**A**) Representative whole-cell voltage-clamp recordings; (**B**) normalized mEPSC frequencies; (**C**) cell membrane capacitances, and (**D**) resting membrane potentials from mESN cultures treated with vehicle, *wt* BoNT/C1 (400 fM, equivalent to 2 mouse LD_50_ units/mL), or BoNT/C1 *ad* (100 nM, equivalent to 0.2 mouse LD_50_ units/mL) for 20 h. Normalized data are from 12–16 recordings per treatment, and are presented as mean ± SEM; ***p < 0.001.

Previous work has demonstrated that intoxication by *wt* BoNT/A–/G impairs spontaneous synaptic transmission in synaptically networked cultures of mouse embryonic stem cell-derived neurons (mESNs)^15^,^16^. To determine if BoNT/C1 *ad* affects synaptic function *in situ*, synaptically active cultures of mESNs were treated with vehicle, *wt* BoNT/C1 (400 fM, equivalent to 2 mouse LD_50_ units/mL), or BoNT/C1 *ad* (100 nM, equivalent to 0.2 mouse LD_50_ units/mL) for 20 h, and spontaneous miniature excitatory post-synaptic current (mEPSC) frequencies were quantified from whole-cell voltage-clamp recordings (Fig. 6A, B). In cultures treated with *wt* BoNT/C1, mEPSC frequencies were reduced approximately 80% compared to vehicle-treated controls, indicating that *wt* BoNT/C1 treatment blocked spontaneous release of neurotransmitter. In contrast, treatment with a 250,000-fold higher concentration of BoNT/C1 *ad* did not affect mEPSC frequency, suggesting that BoNT/C1 *ad* did not impair neurotransmitter exocytosis from the pre-synaptic compartment. Furthermore, neither *wt* BoNT/C1 nor BoNT/C1 *ad* altered passive membrane properties such as cell membrane capacitance (Fig. 6C) or resting membrane potential (Fig. 6D). These findings suggest that prolonged incubation with 100 nM BoNT/C1 *ad* does not acutely interfere with action potential propagation or neurotransmitter release, indicating that BoNT/C1 *ad* is not cytotoxic, even when administered at relatively high concentrations.

### Absence of BoNT/C1 *ad* cytotoxicity *in vitro*

**Figure 7:**
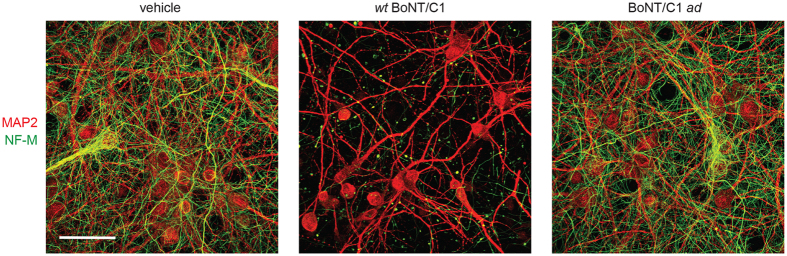
BoNT/C1 *ad* does not cause axonal degeneration. 11–13 day cultures of E19 rat hippocampal, cortical, and ventricular zone neurons were treated with vehicle, *wt* BoNT/C1 (0.5 nM, equivalent to 2,508 mouse LD_50_/mL), or BoNT/C1 *ad* (50 nM, equivalent to 0.1 mouse LD_50_ units/mL) for 48 h and stained with NF-M (green) and MAP2 (red). Scale bar = 50 μm, n = 4 per treatment.

At high doses, *wt* BoNT/C1 causes acute degeneration of cultured neurons, which is believed to result from the dual cleavage of SNAP-25 and Syx1^17^. To evaluate the cellular effects of intoxication by the catalytically inactive BoNT/C1 *ad*, 11–13 d cultures of E19 rat hippocampal, cortical, or ventricular zone neurons were exposed to vehicle, BoNT/C1 *ad* (100 nM, equivalent to 0.2 mouse LD_50_ units/mL), or *wt* BoNT/C1 (500 pM, equivalent to 2,508 mouse LD_50_ units/mL) for 48 h, and the integrity of axonal and dendritic processes were evaluated by immunocytochemistry. Cultures treated with BoNT/C1 *ad* retained morphologically normal axodendritic arbors, as demonstrated by contiguous neurofilament staining of axons and by the presence of dendrites (Fig. 7). In contrast, neurons treated with *wt* BoNT/C1 exhibited fragmented and degenerated axons, while still retaining normal-appearing dendritic arbors, a pattern of degeneration that is consistent with anterograde toxicity^18^. Collectively, these data indicate that BoNT/C1 *ad* is more than 200-fold less cytotoxic than the *wt* toxin.

### BoNT/C1 *ad* LC co-localizes with synaptic proteins

**Figure 8:**
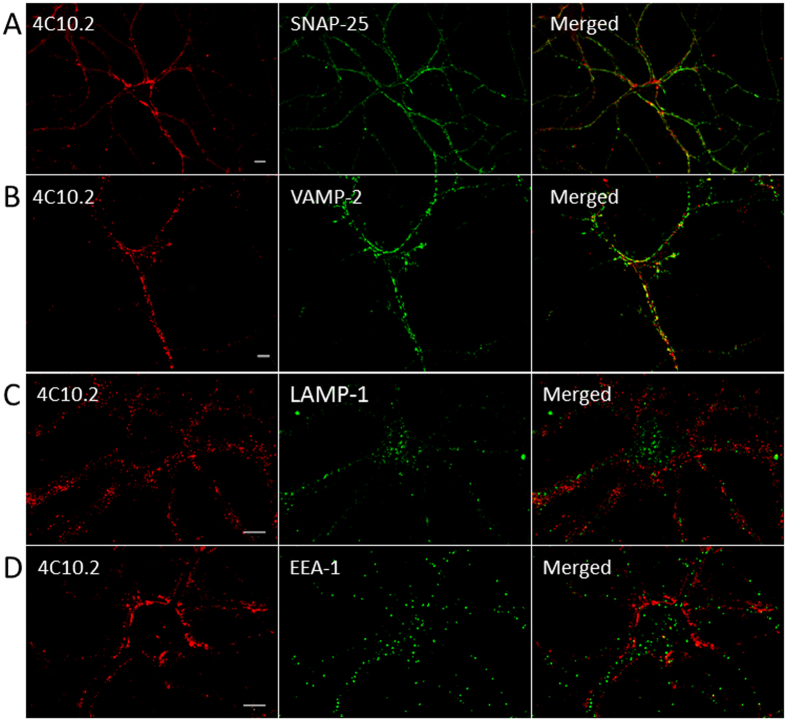
BoNT/C1 *ad* co-localizes with synaptic markers after neuronal internalization. Cultures of 14-DIV rat hippocampal neurons were exposed to 25 nM BoNT/C1 *ad* (probed with anti-human IgG 4C10.2) for 16 hours. Cells were prepared for immunocytochemistry, and analyzed using confocal microscopy for the light chain of BoNT/C1 *ad* (red staining) and for pre-synaptic markers SNAP-25 (**A**) and VAMP-2 (**B**), lysosomal marker LAMP-1 (**C**), and early endosomal marker EEA-1 (**D**) (green staining). Yellow color in merged images shows co-localization of BoNT/C1 *ad* LC and the specific marker. Bar = 10 μm.

The cellular target of *wt* BoNT/C1 is the presynaptic compartment, where the LC associates with and cleaves the neuronal SNARE proteins SNAP-25 and Syx1. To confirm that BoNT/C1 *ad* undergoes neuronal uptake and trafficking in a fashion similar to *wt* BoNT/C1, we evaluated its co-localization with presynaptic proteins in cultured neurons. Primary rat cortical or hippocampal neurons were exposed to 25 nM BoNT/C1 *ad* for 24 hours. Immunocytochemical analysis showed that the light chain of BoNT/C1 *ad* co-localized with pre-synaptic SNARE proteins SNAP-25 and VAMP-2 (Fig. 8A, B). To confirm that BoNT/C1 *ad* LC was not associated with endosomal vesicles and likely destined for degradation, we co-stained cell cultures exposed to BoNT/C1 *ad* with mAbs against EEA-1 (early endosome antigen 1) and lysosomal marker LAMP-1 (lysosome-associated membrane protein 1). There were some minor instances of co-localization with EEA-1, consistent with the role of the early endosome as a transient reservoir associated with BoNT trafficking, but the overall distribution patterns were distinct, indicating that the majority of BoNT/C1 *ad* did not accumulate in early or late endosomes (Fig. 8C, D).

### Detection of BoNT/C1 *ad* at the NMJ *in vivo*

**Figure 9:**
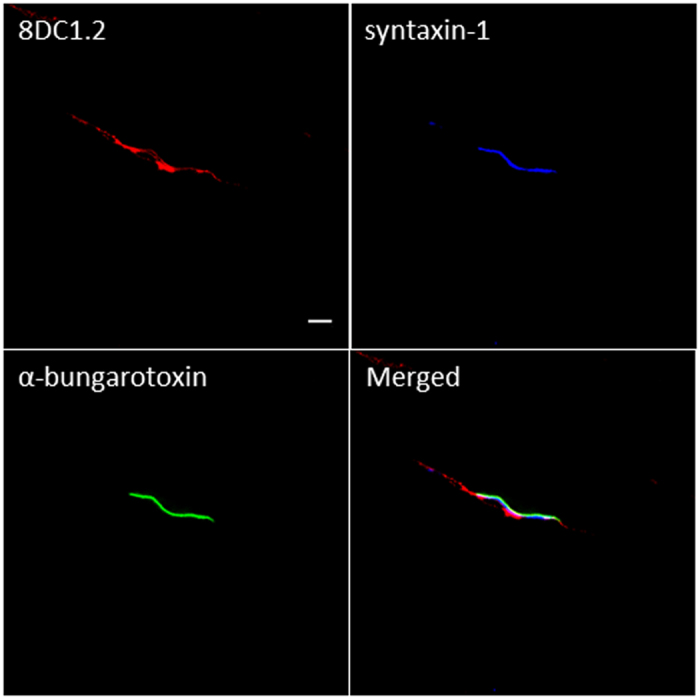
After systemic administration, BoNT/C1 *ad* specifically accumulates at the neuromuscular junction and co-localizes with synaptic proteins. Six week-old mice were injected *ip* with 0.4 mg/kg of BoNT/C1 *ad*. 24 hours after systemic injection, mice were euthanized, and diaphragms were isolated and prepared for staining. Tissue was stained with mAbs raised against Syx1 (pre-synaptic marker), BoNT/C1 HC (probed with anti-human IgG 8DC1.2), and α-bungarotoxin (post-synaptic marker), and analyzed by confocal microscopy. Bar = 10 μm.

The physiologic target of circulating BoNTs is the pre-synaptic compartment of peripheral motor neurons, autonomic synapses, and preganglionic neurons. To determine if *in vivo* trafficking of BoNT/C1 *ad* following systemic exposure was similar to that of *wt* BoNT/C1, 6 week-old CD-1 female mice were injected *ip* with 0.4 mg/kg of BoNT/C1 *ad*, and the diaphragm was isolated for co-localization studies 24 h after injection. Processed diaphragms were stained with antibodies against BoNT/C1 heavy chain (8DC1.2) and Syx1 (as a motor nerve terminal marker). The post-synaptic motor endplate was stained with alpha-bungarotoxin. Microscopic examination of the stained tissues indicated that BoNT/C1 *ad* closely matched localization of both Syx1 and alpha-bungarotoxin staining, confirming that BoNT/C1 *ad* traffics to the NMJ *in vivo* after systemic administration (Fig. 9).

### BoNT/C1 *ad* is not associated with autophagosome marker

**Figure 10:**
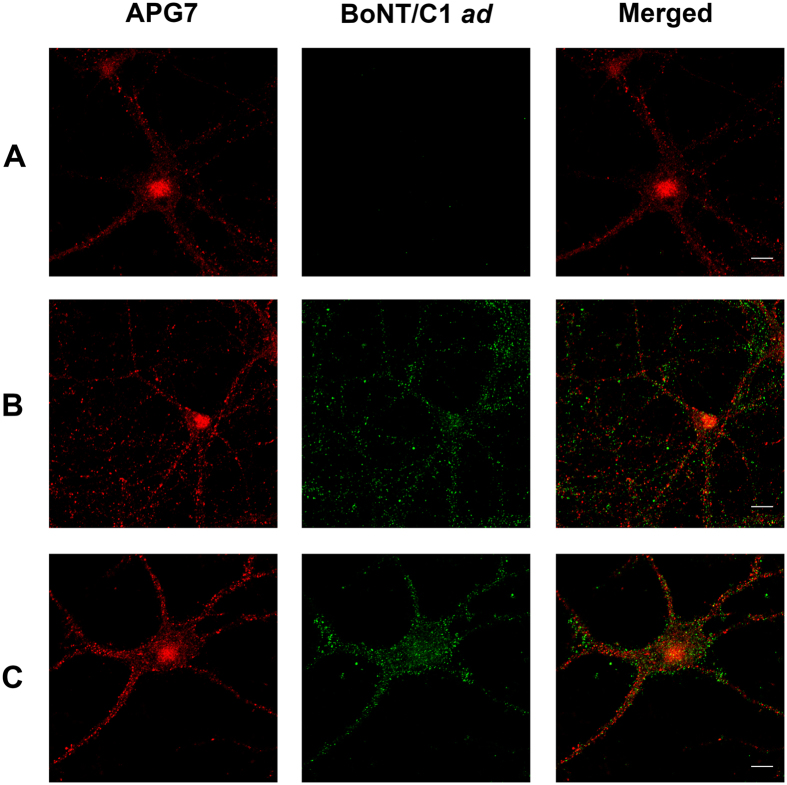
Immunostaining of neurons treated with BoNT/C1 *ad* and chased with medium. Cultures of rat hippocampal neurons, 14 days after plating, were exposed to 25 nM BoNT/C1 *ad* for 48 hours, chased with fresh medium for 48 or 72 hours, prepared for immunocytochemistry as described in Materials and Methods, and analyzed using confocal microscopy. Cells were stained for autophagy marker APG7 (red) and for the heavy chain of BoNT/C1 *ad* (green). Panel **A**: cells not treated with BoNT/C1 *ad,* Panel **B**: 48 hours chase after exposure to BoNT/C1 *ad*, Panel **C**: 72 hours chase after exposure to BoNT/C1 *ad*. Bar = 10 μm.

To explore a possible relationship between the toxicity exhibited by BoNT/C1 *ad in vivo* and the recycling of internalized protein through autophagosome as a compensatory mechanism for the potential transient overload of the endosomal/lysosomal pathway, we also examined the pattern of co-localization and intensity of autophagy-related protein 7 (APG-7), an autophagy marker, in neuronal cultures treated with 25 nM BoNT/C1 *ad* for various times of incubation and chase with fresh medium. Neuronal cultures were treated with 25 nM BoNT/C1 *ad* for 24 hours and chased with fresh 50% conditioned medium for 48 and 72 hours (Fig. 10). Neurons not treated with BoNT/C1 *ad*, or treated with BoNT/C1 *ad* for various time periods, and not chased, or chased after treatment with fresh medium for various time periods showed the same intensity and distribution pattern of APG-7. No significant co-localization of the BoNT/C1 *ad* heavy chain with APG-7 was detected.

## Discussion

The studies presented here are part of our ongoing effort to engineer recombinant derivatives of BoNTs capable of delivering a therapeutic agent to the neuronal cytoplasm by exploiting the trafficking pathways used by native *wt* BoNT. Here, we designed, expressed, purified, and evaluated a derivative of BoNT serotype C1 (BoNT/C1 *ad*) rendered atoxic through amino acid substitutions E_238_ > A; H_241_ > G; and Y_383_ > A. We confirmed that BoNT/C1 *ad* targets pre-synaptic neuronal terminals *in vitro* and *in vivo*. We found that BoNT/C1 *ad* maintained the structural integrity of *wt* BoNT/C1, and trafficked to the pre-synaptic compartment at the NMJ of the diaphragm after systemic administration. BoNT/C1 *ad* light chain co-localized with presynaptic SNARE proteins, and failed to co-localize with endosomal, lysosomal or autophagosomal markers, suggesting that it traffics to the presynaptic cytosol without being sequestered in a vesicular compartment or destined for rapid degradation. Using an *in vivo* mouse bioassay, we determined the LD_50_ of the BoNT/C1 *ad* to be 5 mg/kg. No metalloprotease activity was detected for BoNT/C1 *ad* in multiple *in vitro* assays over a wide range of concentrations, even though in some of these assays the concentration of the protein exceeded the concentration that was associated with symptoms *in vivo*. It was also found that BoNT/C1 *ad* was approximately 5 × 10^6^-fold less toxic than *wt* BoNT/C1^10^. Extrapolations from *in vivo* mouse bioassay data suggested that the high-end dose that can be safely injected into an average (70 kg) human without causing side effects is equal to 56 milligrams. Collectively, these findings indicate that BoNT/C1 *ad* may be useful as a molecular vehicle for drug delivery to the cytosolic pre-synaptic compartment of neurons.

The decision to switch from BoNT/A1 to BoNT/C1 as the precursor for development of a neuronal delivery vehicle was influenced by several factors. BoNT/A1 *ad* would be ineffective as a delivery vehicle for a therapeutic against *wt* BoNT/A1, which is the serotype responsible for nearly 50% of clinical cases of botulism in the United States from 2004–2014^19^. This is because any anti-BoNT/A1 therapeutic moiety (small molecule inhibitors, peptides, or antibodies) intended to bind and inactivate the *wt* BoNT/A1 LC in the neuronal cytoplasm would also recognize the delivery vehicle itself, and likely would render the therapeutic moiety inactive. *wt* BoNT/C1 rarely causes human botulism^20^, and therefore, there are no similar restrictions on use of the *wt* BoNT/C1-based vehicle for delivery of anti-BoNT therapies against major human BoNT pathogens, such as serotypes A, B and E. In contrast to BoNT/A1 *ad*, a BoNT/C1 *ad*-based delivery vehicle could deliver a therapeutic moiety against these serotypes into the neuronal cytoplasm without the danger of self-recognition and inactivation.

Cellular entry of *wt* BoNT/C1 employs a mechanism that is different from the six other BoNT serotypes. As shown in several reports, the presence of Sia-5 and Sia-7 gangliosides on the neuronal plasma membrane appears to be sufficient for BoNT/C1 internalization^21^. Although attempts to identify a protein-based receptor for BoNT/C1 have failed^22^,^23^,^24^, evidence of increased toxicity following potassium stimulation of neuronal cultures suggests that entry may be enhanced by cell surface components that are involved in activity-dependent synaptic endocytosis^21^,^23^,^25^. *wt* BoNT/C1 is able to enter neurons that were previously intoxicated with *wt* BoNT/A1, which is consistent with the hypothesis that these two serotypes use different mechanisms of internalization^26^, and suggests that BoNT/C1 *ad* can be used as a molecular vehicle to deliver *wt* BoNT/A1-inactivating therapeutic entities to the neuronal cytoplasm of *wt* BoNT/A1-intoxicated neurons, an important feature, because the therapeutic cargo will reside in the same subcellular compartment as the active toxin.

The idea that BoNT can be used as a vehicle for delivery of therapeutic cargo to neurons has a long history. From the structural prospective, the best option for the placement of therapeutic cargo (small molecule, peptide or protein) can be achieved through the linkage of the cargo to the N-terminus of metalloprotease-inactivated LC of the toxin heterodimer (LC-fused cargo)^5^. In our design of BoNT/C1 *ad*, nine additional amino acids are placed at the N-terminus of the atoxic light chain. The primary purpose of these extra amino acids is spatial separation of the affinity tag (His tag) from the rest of the molecule. In our earlier experiments, we found that when this tag was directly linked to the first proline residue of the BoNT light chain, accessibility of the affinity tag was compromised (unpublished observation). These nine amino acids can be viewed as mini-cargo that we were able to deliver to the intraneuronal cytoplasm along with the rest of the light chain. The HC of BoNT does not translocate into the cytoplasm, and thus, placement of cargo in this location is unsatisfactory. Moreover, placement of large cargo at the C-terminus of the HC could interfere with the recognition of the cognate receptor, and could abrogate the ability of the molecule to deliver the cargo to the neuronal cytoplasm. Placement of large cargo at the C-terminus of the LC or N-terminus of the HC could interfere with the tight organization of the LC-HC macromolecule, and could disrupt the disulfide linkage of the native heterodimer. Thus, if the designed molecule is internalized and processed similar to *wt* BoNT, then LC-fused cargo should escape the early endosome and be translocated into the neuronal cytoplasm. Specific neuronal delivery of the therapeutic cargo through this *wt* BoNT mechanism is expected to circumvent problems associated with impermeability of the cellular plasma membrane, and toxicity due to drug reaching off-target cells.

BoNT/C *ad* LC is delivered to the pre-synaptic compartment of neurons (Fig. 8). This is in contrast to reports in which recombinant BoNT derivatives delivered to neurons have become entrapped in endosomes with little or no co-localization with SNARE proteins, suggesting that the BoNTs did not gain complete access to the cytosolic compartment^27^,^28^,^29^,^30^,^31^. There is, however, at least one report in which the authors convincingly show delivery of large protein molecules to the neuronal cytoplasm as a fusion with full-length recombinant BoNT/D^1^.

BoNT/C1 *ad* has an improved therapeutic margin, compared to BoNT/A *ad*^7^. Mice injected with high but sub-lethal (2 mg/kg) doses of BoNT/C1 *ad* (Table 1) exhibited symptoms of neuromuscular disruption (reduced mobility, decrease in respiratory patterns, and wasp-like waist) that resolved within 72 hours after challenge. One possible explanation for the observed symptoms may be due to the ability of the light chain of BoNT/C1 *ad* to accumulate in the presynaptic compartment and to bind to its native endogenous substrates—SNAP-25 and Syx1. Even though cleavage of the substrates is disabled, binding of BoNT/C1 *ad* to the substrates may interfere with their assembly into the SNARE protein fusion complex necessary for synaptic exocytosis. However, there is also considerable experimental evidence against this explanation: 1) data from unaltered spontaneous neurotransmitter release presented here suggest that SNARE sequestration is not a major factor for *in vivo* toxicity of BoNT/C1 *ad*; 2) the transient characteristic of the toxicity did not correlate with the stability of the light chain of BoNT/C1 *ad* in neuronal cytoplasm; 3) the comparative molar ratio of BoNT/C1 *ad* light chain to SNAP-25, approximately 1 molecule of the BoNT/C1 *ad* light chain to over 100 molecules of SNAP-25, makes it unlikely that BoNT/C1 *ad* light chain could be responsible for the mechanical sequestration of SNARE proteins in the absence of cleavage.

BoNT/C1 *ad* light chain has a long lasting persistence in neuronal cytoplasm. In pulse-chase experiments, BoNT/C1 *ad* light chain was detected in whole cell lysates for as long as eight days after exposure, which is similar to our observation for the stability of the light chain of BoNT/A1 *ad*^7^. Unlike the slow decline of the intracellular concentration of BoNT/A *ad* light chain starting at day 7 of chase^7^, we did not detect significant changes in intracellular concentration for the light chain of BoNT/C1 *ad* between days 1 and 8 of chase (Fig. 5). Alternatively, the observed *in vivo* toxicity of BoNT/C1 *ad* may result from overload of intracellular protein homeostasis systems. However, the data presented here seem to rule out this possibility as well, in that the *in vivo* toxicity of BoNT/C1 *ad* was not a consequence of transient overload of the endosomal/lysosomal pathway leading to recycling of the protein *via* an alternative stress-response system such as autophagy (Fig. 10).

Ganglioside-mediated cellular entry of BoNT/C1 may provide yet another clue for the transient toxicity of BoNT/C1 *ad in vivo*. Gangliosides have a natural predisposition to lateral segregation within membranes, and represent an important integral component of membrane microdomains (or lipid rafts) that are enriched with cholesterol^32^. Sphingolipids and gangliosides are important modulators of membrane receptors, ion channels, and downstream signaling pathways. Regulation occurs by different mechanisms, some of which are rather general and others that are receptor and/or ganglioside-specific^32^. Binding of the BoNT/C1 *ad* heavy chain to gangliosides may contribute to their sequestration from interaction with their endogenous partners, and thereby, change the physical properties of the plasma membrane. This sequestration may also lead to transcriptional activation, resulting in increased endogenous *de novo* synthesis of gangliosides, as a compensatory mechanism^33^,^34^. The time frame (24–72 hours) required for disappearance of BoNT/C1 *ad* toxicity *in vivo* is consistent with this hypothesis. However, the exact nature of residual *in vivo* BoNT/C1 *ad* toxicity needs further investigation, not only for understanding the molecular mechanisms, but also from the practical perspective of developing neuronal delivery vehicles with an expanded therapeutic window.

Development of tools aimed at delivering therapeutic moieties to the cytoplasm of neurons is challenging and plagued by numerous side effects, such as cytotoxicity of lipophilic micelle-forming agents and cell-permeabilizing peptides^35^,^36^. Existing viral delivery vectors are taken up by a majority of cell lines, reflecting the lack of specific entry mechanism^37^, and even replication-incompetent viruses pose a long-term risk related to their abilities to 1) integrate into genomic DNA, 2) contribute to the synthesis of proteins other than intended therapeutic cargo with the potential deleterious side effect of unintended expression, and 3) cause cytotoxicity *via* internalization of their integral envelope/nucleocapsid proteins. Use of viral delivery vectors also poses regulatory challenges, especially from the perspective of defining the proper dosage of therapeutic delivered in the form of DNA that needs to be expressed. In this report we have proposed a practical alternative: the use of a relatively simple, multi-domain BoNT molecule as a molecular delivery vehicle. This can have multiple advantages, including simplicity of internalization, absence of cytotoxicity at the high concentrations used (up to 500 nM), high specificity, and functional flexibility (for example, through the introduction of features to modulate intracellular stability and duration of the therapeutic cargo^38^). Although the potential advantages of delivery vectors such as BoNT/C1 *ad* are clear, expression of recombinant BoNT/C1 proteins in properly folded, physiologically active di-chain form has proven technically challenging. In this regard, an early publication^39^ reporting the expression of the BoNT/C1 atoxic protein in *E.coli*, with a design similar to ours, provided no evidence of satisfactory yield, successful processing of the expressed pro-peptide, or whether the expressed protein mimicked the trafficking pattern of the *wt* BoNT/C1.

In summary, the data presented here suggest that BoNT/C1 *ad* provides a promising molecular vehicle for drug delivery to therapeutic targets in the pre-synaptic compartment of neurons. BoNT/C1 *ad* is a neuron-specific non-viral vector capable of targeting the cytoplasm of neurons *in vivo*. Relevant intra-neuronal targets are of interest for multiple neurological diseases, including reversing intoxication with BoNT itself. The generalizability of this approach is under active investigation.

## Materials and Methods

### Reagents/Supplies

Reagents included ammonium bicarbonate (Acros Organics, Cat. 393212500), *D*-desthiobiotin (Sigma-Aldrich, Cat. D1411), *DL-*dithiothreitol (Sigma-Aldrich, Cat. D0632-5G), formaldehyde, 16% solution (Electron Microscopy Sciences, Cat. 15710), formic acid (Thermo Fisher Scientific, Cat. A117-50), *L*-glutathione oxidized (Sigma-Aldrich, Cat. G4376), *L*-glutathione reduced (Sigma-Aldrich, Cat. G4251), imidazole (Thermo Fisher Scientific, Cat. BP305-50), iodoacetamide (MP Biomedicals LLC, Cat. 100351), β-mercaptoethanol (Bio-Rad, Cat. 1610710), polyethylenimine (Sigma-Aldrich, Cat. 408727), 10% Triton X-100 (surfact-Amps X-100, Thermo Fisher Scientific, Cat. 28314), 1X DPBS (Thermo Fisher Scientific, Cat. 21-030-CV), 2% sterile gelatin solution (Sigma-Aldrich, Cat. G1393), Criterion XT 4–12% Bis-Tris gel (Bio-Rad, Cat. 3450124), XT MES Running Buffer (Bio-Rad, Cat. 1610789), 4x Laemmli sample buffer (Bio-Rad, Cat. 1610747), Coomassie Blue R-250 stain solution (Teknova, Cat. C1050), 4–12% Bis-Tris NuPAGE gel (Thermo Fisher Scientific, Cat. NP0323BOX), 20x NuPAGE MOPS SDS running buffer (Thermo Fisher Scientific, Cat. NP000102), 4x NuPAGE LDS sample buffer (Thermo Fisher Scientific, Cat. NP0007), SimplyBlue SafeStain (Thermo Fisher Scientific, Cat. LC6060), BCA assay kit (Thermo Fisher Scientific, Cat. 23227), nitrocellulose membranes (Bio-Rad, Cat. 162–0168), thick blot paper (Bio-Rad, Cat. 1703956), mouse serum albumin (Albumin Bioscience, Cat. 2601), normal goat serum (Thermo Fisher Scientific, Cat. 16210–072), SuperSignal West Pico chemiluminescent substrate (Thermo Fisher Scientific, Cat. 34080), α-chymotrypsin (Sigma-Aldrich, Cat, C4129-250MG), endoproteinase Asp-N (Sigma-Aldrich, Cat. 13439321), trypsin, sequencing grade (Promega, Cat. V511A), BL21 (DE3) CodonPlus-RIL cells (Agilent Technologies Cat. 230245), pRK793 6-His-TEV protease plasmid (Addgene, Cat. 8827), Neurobasal Medium (Thermo Fisher Scientific, Cat. 21103049), B27 supplement (Thermo Fisher Scientific, Cat. 17504044), BrainBits NbActiv4 medium (BrainBits, Cat. Nb4-500), SF-900 II serum-free medium (Thermo Fisher Scientific, Cat. 10902088), anti-β-actin mouse IgG2a (Sigma-Aldrich, Cat. A5316), anti-EEA-1 mouse IgG (BD Bioscience, Cat. 610457), anti-HA 3F10 rat IgG (Sigma-Aldrich, Cat. 11867423001), anti-LAMP-1 rabbit IgG (EMD Millipore, Cat. AB2971), anti-LC3B rabbit IgG (Abcam, Cat. ab48394), anti-APG7 rabbit IgG (Santa Cruz Biotechnology, Cat. 33211) anti-MAP2 chicken IgY (Abcam, Cat. ab5392), anti-neurofilament M rabbit IgG (EMD Millipore, Cat. ab1987), anti-SNAP-25 mouse IgG (Synaptic Systems, Cat. 111011), anti-syntaxin-1 mouse IgG (Synaptic Systems, Cat. 110011), anti-rat Tau mouse IgG2b (BD Bioscience, Cat. 610672), anti-VAMP-2 mouse IgG (Synaptic Systems, Cat. 104211), Alexa Fluor 555 goat anti-human IgG (Thermo Fisher Scientific, Cat. A-21433), Alexa Fluor 555 goat anti-mouse IgG2b (Thermo Fisher Scientific, Cat. A-21422), Alexa Fluor 647 goat anti-mouse IgG2b (Thermo Fisher Scientific, Cat. A-21242), Alexa Fluor 647 goat anti-rabbit IgG (Thermo Fisher Scientific, Cat. A-21245), Alexa Fluor 488 goat anti-human IgG (Thermo Fisher Scientific, Cat. A-11013), Alexa Fluor 488 conjugated alpha-bungarotoxin (Thermo Fisher Scientific, Cat. B-13422), Prolong Gold DAPI mounting media (Thermo Fisher Scientific, Cat. P36961), botulinum neurotoxin type C (Metabiologics), Ni^2+^-NTA Sepharose fast flow (IBA GmbH, Cat. 2-3206-025), StrepTactin Sepharose fast flow (IBA GmbH, Cat. 2-1208-025), HiLoad 26/600 Superdex^™^ 200 pg gel filtration column (GE Healthcare Life Sciences, Cat. 28989336), and 0.22 micron filters (EMD Millipore, Cat. UFC30GV0S). All basic chemicals were purchased either from Thermo Fisher Scientific (Waltham, MA), or from Sigma Aldrich (St. Louis, MO), unless indicated otherwise.

### Methods Statement

All methods were performed in accordance with the relevant guidelines and regulations.

### Ethics Statement

Experiments involving animals were conducted with the approval of the City College of City University of New York (CCNY), or the Tufts Cummings School of Veterinary Medicine (TCSVM) Institutional Animal Care and Use Committee. Both animal facilities are maintained in accordance with the Animal Welfare Act, United States Department of Agriculture Regulations (9 CFR, Parts 1, 2, and 3), and the Guide for the Care and Use of Laboratory Animals (National Academy Press, Revised 2011). CCNY has a currently approved Animal Welfare Assurance Agreement (No. A3733-01) with the NIH Office for Protection from Research Risks.

### Animals

Eight week old, CD-1 female mice (Charles River’s Laboratories) were housed five per cage in a barrier facility, and were maintained on a 12-hour light/dark cycle (7 AM to 7 PM) with *ad libitum* access to food and water. The average weight of mice used for determination of BoNT/C1 *ad* toxicity *in vivo* was 23.2 grams. The average weight of mice used for determination of *wt* BoNT/C1 potency *in vivo* was 25.8 grams.

### Expression and processing of BoNT/C1 *ad*

The gene for BoNT/C1 *ad* was engineered with tRNA bias typical for the *Sf9* cell translational machinery and synthesized *de novo*. The full-length BoNT/C1 *ad* DNA was incorporated into recombinant baculovirus as described^5^, and the protein was expressed as a secreted pro-peptide, in accordance with CDC regulations (GenBank accession number for BoNT/C1 *ad* is KX496548). Protein was expressed and purified using the same baculovirus methodology described for BoNT/A1 *ad*^5^. SF 900II medium supernatants containing secreted BoNT/C1 *ad* pro-peptide were collected. The BoNT/C1 *ad* was sequentially purified by tandem affinity chromatography on Ni^2+^-NTA and StrepTactin Sepharose fast flow resins. Protein eluted from the Ni^2+^-NTA resin with buffer containing 250 mM imidazole was loaded on StrepTactin sorbent, and after several washes was eluted with 5 mM *D*-desthiobiotin. Aliquots representing loading material, flow through, washes, and eluates from both sorbents during the purification process were loaded on reducing SDS PAGE, separated, and stained with Coomassie Brilliant Blue R250 (Fig. 1A). A homogeneous pro-peptide, eluted from StrepTactin resin (lane 13, Fig. 1A) was concentrated, dialyzed against 40% glycerol/PBS and stored at −80 °C. For generation of the BoNT/C1 *ad* heterodimer and removal of the affinity tags, the purified pro-peptide was treated with 6-His-TEV protease (1 mg 6-His-TEV per 5 mg of BoNT/C1 *ad* pro-peptide) for 48 hours at 25 °C in the presence of 3 mM reduced/0.3 mM oxidized glutathione, to provide necessary reducing potential for TEV activity without contributing to the reduction of the disulfide bridge between LC and HC of BoNT/C1 *ad* heterodimer. 6-His-TEV protease was expressed in the laboratory using methods adapted from^40^. Briefly, expression plasmid pRK793, encoding 6-His-TEV protease, was transformed into BL21 (DE3) CodonPlus-RIL cells, and grown in 1.5L cultures producing 5 mL cell paste per L. The yield of purified protease was about 4.5 mg/L of culture. Analysis of TEV cleavage after 48 hours of incubation with the pro-peptide showed that more than 95% of the BoNT/C1 *ad* was cleaved into heterodimer (Fig. 1B). The removal of 6-His-TEV was performed by another round of Ni^2+^-NTA affinity chromatography; BoNT/C1 *ad* heterodimer remained in the flow through fraction and initial washes with low imidazole (Fig. 1C). After concentration of the fractions containing BoNT/C1 *ad* heterodimer, the protein was subject to final chromatography on HiLoad 26/600 Superdex^™^ 200 pg gel filtration column for removal of aggregates and low molecular weight contaminants. The protein from the major peak (Supplemental Figure S2) was concentrated, dialyzed against 40% glycerol/PBS, sterile filtered through a 0.22 micron filter, and concentration was determined by BCA assay. Final concentration of the heterodimer was normalized to 10 mg/mL, and the protein was aliquoted and stored at −80 °C.

### Proteomic characterization of BoNT/C1 *ad* heterodimer

#### PAGE separation

A 25 μg protein sample in 5 μL of a solution consisting of 40% glycerol, 100 mM NaCl, 25 mM sodium phosphate, pH 7.5 was mixed with 7 μL water and 4 μL 4x SDS PAGE sample loading buffer, and heated at 90 °C for 10 min. The sample was split into equal aliquots and loaded in triplicate into three wells of a 4–12% Bis-Tris NuPAGE gel. Gel electrophoresis was performed at a constant voltage of 180 V for 20 min in NuPAGE MOPS SDS running buffer. The gel was rinsed three times with 100 mL water for 5 min and stained with 20 mL of SimplyBlue SafeStain staining solution for 1 h at room temperature followed by washing four times with 100 mL water for 10 min.

#### In-gel proteolytic digestion

The gel band containing BoNT/C1 *ad* from each of the sample wells was excised in 1 mm cubic pieces and pooled in a 1.5 mL Eppendorf tube. Each sample was reduced and alkylated by sequential incubation in 150 μL of 20 mM *DL-*dithiothreitol at 60 °C for 1 h, followed by incubation in 150 μL of 55 mM iodoacetamide in the dark room temperature for 45 min; reagent solutions were removed after each step. Gel pieces were de-stained twice in 300 μL of 1:1 (*v:v*) acetonitrile/100 mM ammonium acetate solution for 1 min on a shaker, and dehydrated in 150 μL acetonitrile for 10 min. After acetonitrile removal, the gel was consecutively digested overnight at 37 °C in 150 μL of freshly prepared protease solutions made up in 50 mM ammonium bicarbonate; trypsin, chymotrypsin and flavastacin (Asp-N) with protease concentrations at 0.02 μg/μL. Supernatant from each digestion was collected in a separate tube and vacuum-centrifuged to dryness. Tryptic, chymotryptic, and Asp-N digest were re-suspended respectively in 80, 60, and 40 μL of 5/95/0.1 acetonitrile/water/formic acid (*v:v*) for nanoLC-MSMS analysis.

#### nanoLC-HRMSMS analysis

The digests were analyzed in triplicate on a nanoLC-MSMS system composed of a Thermo nLC EASY1000 interfaced with a Thermo Fisher Scientific Fusion Orbitrap mass spectrometer. There was a range of peptide concentrations in these digests; trypsin digests were the most concentrated, and Asp-N digests were the least concentrated. Trypsin (2 μL), chymotrypsin (3 μL), and AspN (4 μL) digest samples were loaded on a 100 μm id × 20 mm × 5 μm 200 Å AQC18 trap column. Trapped peptides were eluted and separated on a 75 μm id × 18 cm × 5 μm 100 Å AQC18 analytical column using a 120 min nanoUPLC run. Reversed phase chromatography was performed using 0.1% formic acid in water and 0.1% formic acid in acetonitrile as mobile phases A and B, respectively. Mass spectrometric data were collected using a 3 sec top-speed data-dependent acquisition (DDA) method in which full MS scans were collected on an Orbitrap detector at a resolving power of 120,000 over a m/z 350–1500 range. Peptide precursors were isolated for collision induced dissociation (CID), and MSMS spectra from the ion trap were collected using a rapid scan rate.

#### Data processing

The acquired data were processed with MaxQuant software version 1.5.3.30, and searched against a sequence database for BoNT/C1 *ad* light and heavy chains to identify peptides with full-specific trypsin cleavage or semi-specific chymotrypsin and AspN cleavages. Using full scan spectra, peptide matches were performed employing a mass tolerance of 10 ppm, and a 0.5 Da window was used to isolate product ions in MSMS scans. Peptides were identified with a false discovery rate (FDR) of 0.01%.

### Mouse Lethality Assay

BoNT/C1 *ad* was diluted in 1x DPBS with 0.2% gelatin. Mice received injections into the intraperitoneal cavity of a defined dose of BoNT/C1 *ad* diluted in 250 μL of this solution. Clinical observations of botulism toxemia were scored 3 times a day for up to 5 days.

### MS studies of SNARE cleavage

A sample containing 10 μg of BoNT/C1 *ad* (equivalent to 6.7 mouse LD_50_) was added to BoPeptide substrate Ac-VKYNIDEAQNKAS(Orn)MGIRRR-NH2 (SubC) (where Orn is ornithine) in the presence of reaction buffer as previously described^41^ in a final volume of 20 μL and a final SubC concentration of 50 μM. Controls (also in a final volume of 20 μL) consisted of negative control (buffer) and positive controls, including 18.4 pg (equivalent of 1 mouse LD_50_), 184 pg (equivalent of 10 mouse LD_50_), and 100 ng of *wt* BoNT/C1 (equivalent of 5,438 mouse LD_50_). All controls were added to SubC under the same conditions. For detection of the substrate and cleaved products, following a 4 hour incubation at 42 °C, a 2 μL aliquot of each reaction supernatant was added to 18 μL of matrix solution, and a 0.5 μL aliquot of the mixture was pipetted onto one spot of a MALDI plate as described previously^41^. Mass spectra of each spot were obtained by scanning from 900 to 3000 *m/z* in MS-positive ion reflector mode on an Applied Biosystems 5800 Proteomics Analyzer (Framingham, MA). The instrument uses an Nd-YAG laser at 355 nm, and each spectrum was an average of 2400 laser shots. *wt* BoNT/C1 toxin was purchased from Metabiologics (Madison, WI). The potency of the batch was the equivalent of 650,000 mouse LD_50_ per mg of protein. BoPeptide substrate was synthesized by Midwest Bio-tech Inc. (Fishers, IN).

### Western Blot Analysis

Preparation and maintenance of E19 rat cortical and hippocampal neurons were performed as previously described^7^. Neurons were exposed to BoNT/C1 *ad* or *wt* BoNT/C1 kindly provided by Dr. Eric Johnson (University of Wisconsin at Madison) (potency of the batch was equal to 3.23 × 10^6^ mouse LD_50_/mg of protein), as described in Results and figure legends. Neurons were harvested and solubilized; protein was separated and transferred to nitrocellulose membranes as previously described^7^. Membranes were incubated with primary antibodies overnight at 4 °C, and with secondary antibodies for 45 minutes at room temperature. Super Signal West Pico chemiluminescent substrate was used for visualization by autoradiography.

### Analysis of BoNT/C1 *ad* LC stability, concentration, and residual SNAP-25 cleavage in neurons

Approximately 3 × 10^6^ primary rat fetal E19 neurons were isolated and plated according to a described procedure^7^. Neurons were incubated in 50% conditioned medium for 14 days after plating. Incubation was continued for 48 hours either in 50% conditioned medium (control) or in 50% conditioned medium in the presence of 25 nM BoNT/C1 *ad* (equivalent to 0.05 mouse LD_50_ units in the volume used). Medium was then aspirated and cells were washed and chased with 50% conditioned medium without BoNT/C1 *ad* for 24, 96, or 192 hours (192 hours only for controls). After incubation, cells were harvested and solubilized on ice in 500 μL lysis buffer containing 0.5% Triton X-100 and with protease inhibitors, and total protein concentration was measured and normalized. Approximately 55 micrograms of total protein were loaded per lane for each sample, separated on the reduced SDS PAGE, and transferred to a 0.2 μm nitrocellulose membrane. Following transfer, membranes were blocked in 10% fat-free milk supplemented with 5% Normal Goat Serum in TBST (150 mM NaCl, 10 mM Tris-HCl, pH 8.0, 0.1% Tween^®^ 20) at room temperature for 1 hour. Membranes were incubated with primary antibodies overnight at 4 °C and with secondary antibodies 30 minutes at room temperature. Primary antibodies used were anti-BoNT/C1 *ad* LC (4C10.2, provided by Dr. James Marks, UCSF), anti-SNAP-25, and anti-β-actin. Following incubations, blots were washed with TBST 3 times for 5 minutes. Super Signal West Pico chemiluminescent substrate was used for visualization. Li-Cor Odyssey Fc imaging system was used to obtain Western blot images. Li-Cor Image Studio software (version 5.2.5) was used to analyze band signal intensities.

### Electrophysiology

R1 embryonic stem cell lines were obtained from ATCC (Manassas, VA, USA) and differentiated into mouse embryonic stem cell-derived neurons (mESNs) as previously described^13^. Briefly, mESNs were plated at 150,000 cells/cm^2^ in 6 cm dishes coated with polyethylenimine and maintained at 5% CO_2_, 37 °C, and 95% humidity in Neurobasal medium with B27 supplement. Experiments were performed 24 to 26 days after plating. For intoxication with *wt* BoNT/C1 (toxin was purchased from Metabiologics, Madison, WI, batch potency was equivalent to 2 × 10^7^ mouse LD_50_/mg of protein) and BoNT/C1 *ad* proteins were prepared at 100x final concentration in fresh medium and diluted into mESN cultures in a tissue culture glove box (Coy Labs, Grass Lake, MI, USA). Whole-cell patch-clamp electrophysiology was performed to record miniature excitatory post-synaptic currents (mEPSCs) as previously described^15^. mEPSCs were detected from whole-cell recordings using Mini-Analysis v6 with default detection setting from AMPA receptor currents (Synaptosoft Inc., Fort Lee, NJ). Resting membrane potential was determined using the zero-current-clamp method immediately after establishment of whole-cell configuration, and were corrected for a calculated liquid junction potential of 15.6 mV. Membrane capacitance was determined from c-slow values obtained by Heka Patchmaster 2.53 software (Heka, Lambrecht/Pfalz, Germany). Data analysis and graphing were performed in Prism v6.1 (GraphPad software, La Jolla, CA). Averaged mEPSC frequency data were normalized to age- and lot-matched vehicle-treated controls and presented as percent synaptic activity. Significance of differences among means were determined using one-way ANOVA followed by Dunnett’s test; P < 0.05 was considered significant.

### Immunocytochemistry

Fresh E18 rat hippocampal, cortical, and ventricular zone tissues were obtained from BrainBits LLC (Springfield, IL, USA), dissociated according to the manufacturer’s instructions, and plated at a density of 75,000 cells/cm^2^ on polyethylenimine/laminin-coated glass coverslips (Sigma-Aldrich, St. Louis, MO, USA). Neuronal cultures were maintained at 5% CO_2_, 37 °C, and 95% humidity in NbActiv4 medium. Experiments were performed 11 to 13 days after plating (DAP). For intoxication with *wt* BoNT/C1 (Madison, WI; batch potency equivalent to 2 × 10^7^ mouse LD_50_/mg protein) or BoNT/C1 *ad*, proteins were prepared at 100x final concentration in fresh NbActiv4 medium, and diluted into neuronal cultures. Coverslips were then washed with ice-cold DPBS, fixed with ice-cold 4% formaldehyde for 45 minutes at room temperature, and then permeabilized and blocked for 1 h with 0.1% saponin and 3% bovine serum albumin in DPBS (PBSS). Coverslips were then treated with primary antibodies against microtubule associated protein 2 (MAP2, dilution 1:10,000) and neurofilament middle (NF-M, dilution 1:250) in PBSS for 1 h. After washing, coverslips were incubated for 1 h with Alexa-labeled secondary antibodies diluted 1:500 in PBSS. Coverslips were then mounted onto slides with Prolong Gold DAPI mounting media and imaged by confocal microscopy using a Zeiss LSM 700 (Carl Zeiss Inc, Thornwood, New York).

Alternatively, preparation and maintenance of E19 rat cortical and hippocampal neurons were performed as previously described^7^. Cells were exposed to BoNT/C1 *ad* for different times as indicated in figure legends and/or Results. Image scanning was performed on a Nikon LSM 510 confocal microscope, and images were analyzed using Zeiss LSM confocal microscopy software.

### Accumulation of BoNT/C1 *ad* at the diaphragm

Six week-old female CD-1 mice (18 to 22 g, *n* = 5) were injected *ip* with 10 μg of BoNT/C1 *ad* in 250 μL of 0.5% mouse serum albumin in 1x DPBS. Nerve-muscle diaphragm preparations were dissected 24 hours after injection, mounted, and prepared for immunohistochemical staining as previously described^6^. Slides were examined by confocal microscopy using a NIKON LSM-510 microscope equipped with argon and He-Ne lasers, and images were analyzed using Zeiss LSM confocal microscopy software.

### Examination of autophagy compensatory mechanism following internalization of BoNT/C1 *ad*

Preparation and maintenance of E19 rat cortical and hippocampal neurons were performed as previously described^7^. For immunocytochemical studies, 1.5 × 10^5^ cells were plated on cover slips inserted into 6 × 35 mm/well plates in 3 mL medium/well. Neurons (14-day after plating) were exposed to BoNT/C1 *ad* (25 nM) for 48 hours, washed twice with fresh Neurobasal media, and maintained in 50% conditioned media for 48 and 72 hours after washing. Immediately after incubation, cells were washed three times with ice-cold DPBS, fixed with 4% formaldehyde for 15 minutes, and permeabilized with 0.1% Triton X-100 for 5 minutes. After fixation, the permeabilized cells were washed three times with DPBS, blocked for 45 minutes at room temperature with 10% BSA in DPBS, and incubated overnight at 4 °C with primary antibodies: 8DC1.2 (provided by J. Marks, final concentration 1 μg/mL), APG7 (final concentration 0.8 μg/mL). Primary antibodies were diluted in DPBS-NGS. Cells were washed three times with DPBS and incubated for 1 hour with the following secondary antibodies: goat anti-rabbit IgG Alexa Fluor^®^ 555 secondary antibody, or goat anti-human IgG Alexa Fluor^®^ 488 secondary antibody. Final concentration for all secondary antibodies was 0.66 μg/mL. Cells were washed three times with DPBS, and the cover slips were mounted on slides with mounting medium. Image scanning was performed on a Nikon LSM 880 confocal microscope and images were analyzed using Zeiss LSM confocal microscopy software.

## Additional Information

**How to cite this article**: Vazquez-Cintron, E. J. *et al*. Engineering Botulinum Neurotoxin C1 as a Molecular Vehicle for Intra-Neuronal Drug Delivery. *Sci. Rep.* **7**, 42923; doi: 10.1038/srep42923 (2017).

**Publisher's note:** Springer Nature remains neutral with regard to jurisdictional claims in published maps and institutional affiliations.

## Supplementary Material

Supplementary Information

## Figures and Tables

**Table 1:** BoNT/C1 *ad* mouse lethality assay.

Determination of mouse LD_50_ for BoNT/C1 *ad* used in this study		
Dose (mg/kg)	Percent survival (n = 10)	Comments on surviving mice, 5 days observation time
0.04	100	No symptoms for all mice injected. All mice bright, alert, responsive throughout duration of experiment
0.20	100	No symptoms for all mice injected. All mice bright, alert, responsive throughout duration of experiment
0.40	100	No symptoms for all mice injected. All mice bright, alert, responsive throughout duration of experiment
0.80	100	No symptoms for all mice injected. All mice bright, alert, responsive throughout duration of experiment
2.00	90	2 mice bright, alert, responsive throughout duration of experiment; 6 mice bright, alert, responsive with low level severity of abdominal breathing; 1 mouse bright, alert, responsive with high level severity of abdominal breathing. 7 mice with symptoms are fully recovered within 72 hours after injection.
4.00	70	3 mice bright, alert, responsive throughout duration of experiment; 4 mice bright, alert, responsive with high level severity of abdominal breathing. These 4 mice with symptoms are fully recovered within 72 hours after injection.
5.00	50	5 mice bright, alert, responsive with high level severity of abdominal breathing. These 5 mice with symptoms are fully recovered within 72 hours after injection.
6.00	10	2 mice bright, alert, responsive with high level severity of abdominal breathing. These 2 mice with symptoms are fully recovered by day 5 after injection.

Mice were injected *ip* with indicated doses of BoNT/C1 *ad* diluted in 0.2% gelatin-DPBS. Survival was measured three times a day for up to 5 days. The percentage of surviving mice at the end of day 5 is shown.

**Table 2:** Evaluation of intra-neuronal concentration of BoNT/C1 *ad* light chain.

Evaluation of intraneuronal concentration of BoNT/C1 *ad* light chain:	
1.	Molecular weight of BoNT/C1 *ad* LC is 5.2 × 10^4^.
2.	Estimated number of harvested neurons (from 3,000,000 plated) was 3,000,000 ± 500,000 (range:−3,500,000–2,500,000).
3.	Approximate volume of single neuron from rat brain^42^,^43^ was 4,925 ± 2,505 μm^3^ (range: 7,430–2,420 μm^3^).
4.	Approximate volume of all harvested neurons (given by 2 × 3) was 16.025 ± 9.975 μL (range: 26.000–6.050 μL).
5.	Total protein concentration of cell lysate was 3,670 ± 510 μg/mL (range: 4,180–3,160 μg/mL).
6.	Total amount of extracted protein in 500 μL of lysis buffer used for harvesting was 1,835 ± 255 μg (range: 2090–1580 μg).
7.	Amount of BoNT/C1 *ad* LC associated with total extracted protein was 0.278 ± 0.118 ng/μg (range: 0.396–0.160 ng/μg).
8.	Total amount of BoNT/C1 *ad* LC associated with total extracted protein (from 6) was 540.22 ± 287.42 ng (range: 827.64–252.80 ng).
9.	Concentration of BoNT/C1 *ad* LC in the total volume of the harvested neurons (from 4) was 36.81 ± 4.98 ng/μL (range: 41.79–31.83 ng/μL).
10.	Molarity of internalized BoNT/C1 *ad* LC was 705 ± 95 nM (range: 800–610 nM) (molarity calculated from M.W. shown in paragraph 1 and concentration shown in paragraph 9).
