# Supplementary material for: Engineering Botulinum Neurotoxin C1 as a Molecular Vehicle for Intra-Neuronal Drug Delivery
Source: Sci Rep. 2017 Feb 21;7:42923. doi: 10.1038/srep42923 (PMC5318933; doi:10.1038/srep42923)
Supplement: Supplementary Information [file srep42923-s1.pdf]

Supplementary information for the manuscript "Engineering Botulinum Neurotoxin C1 as a Molecular Vehicle for Intra-Neuronal Drug Delivery" by Edwin J. Vazquez-Cintron, Phillip H. Beske, Bao Q. Tran, Jonathan M. Oyler, Elliot J. Glotfelty, Christopher A. Angeles, Luis Tenezaca, Aurelia Syngkon, Jean Mukherjee, Suzanne R. Kalb, Philip A. Band, Patrick M. McNutt, Charles B. Shoemaker and Konstantin Ichtchenko

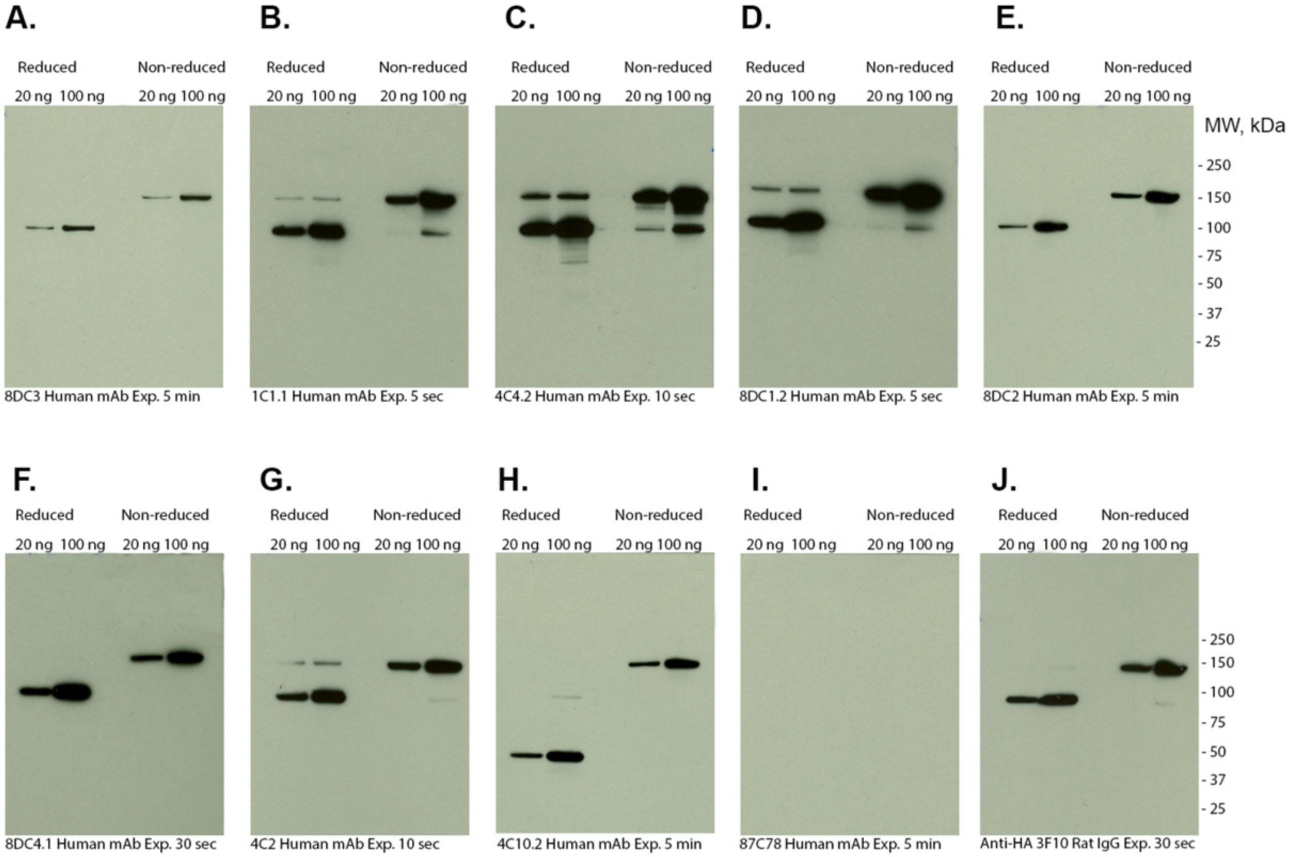

Supplemental Figure S1. Characterization of specificity and sensitivity of the available antibodies against BoNT/C1 *ad* (Western blot analysis). Samples of BoNT/C1 *ad* (20 or 100 ng per lane) were loaded under reducing or non-reducing conditions, separated by SDS-PAGE, and transferred to nitrocellulose membranes. Western blots were probed with human monoclonal mAbs raised against different epitopes/domains of *wt* BoNT/C1 heterodimer (kindly provided by Drs. Jianlong Lou and James Marks, UCSF) (Panels A-I), or commercially available mAb against HA tag (Roche) (Panel J). Western blots were developed with SuperSignal West Pico chemiluminescent substrate. Exposure times are indicated beneath each panel. Molecular weight markers are shown on the right.

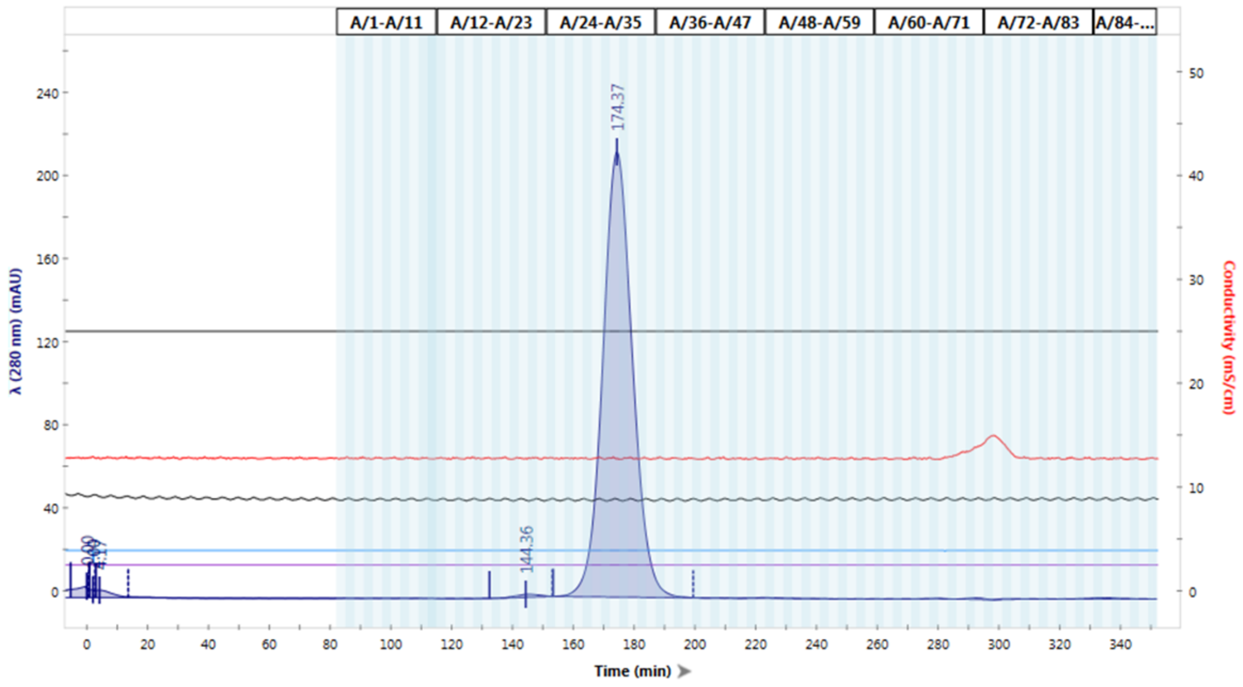

Supplemental Figure S2. Final step of BoNT/C1 *ad* heterodimer purification by gel filtration chromatography. Consolidated fractions containing BoNT/C1 *ad* heterodimer (approximate protein concentration, 30 mg/mL) were loaded in on HiLoad 26/600 Superdex™ 200 pg gel filtration column for removal of aggregates and low molecular weight contaminants. Instrument – NGC Chromatography System (Bio-Rad); Isocratic buffer composition – 100 mM NaCl, 25 mM sodium phosphate, 10% glycerol, final pH 8.0; Sample volume loaded – 1 mL; Flow rate – 1 mL/min; UV detector wavelength – 280 nm; Total time of cycle – 360 min; Fraction size – 3 mL; Void volume of the column – 120 mL; Total bed volume of the column – 330 mL. Protein aggregates are seen as a small peak visible at 144 min (UV detector). BoNT/C1 *ad* heterodimer is seen as a peak at 174 min, consistent with characteristics of the pre-calibrated column, as a BoNT/C1 *ad* monodispersed heterodimer with MW around 150 kDa (dark blue line on UV detector). Low molecular weight impurities are seen at about 290 min as a change in conductivity of the buffer (red line) but no absorbance at 280 nm.
